# The Appendix Is Not Always Alone in Amyand’s Hernia

**DOI:** 10.7759/cureus.12302

**Published:** 2020-12-26

**Authors:** Sherif Monib, Hany F Habashy

**Affiliations:** 1 Breast Surgery, West Hertfordshire Hospitals NHS Trsut, St. Albans and Watford General Hospitals, London, GBR; 2 Surgical Oncology, Fayoum University, Faculty of Medicine, Fayoum, EGY

**Keywords:** congenital inguinal hernia - amyand's hernia- caecum- appendix

## Abstract

An inguinal hernia is one of the most common surgical conditions accounting for both elective as well as acute admissions. Amyand’s hernia is a rare entity of inguinal hernia, which represents a very small percentage of all inguinal hernias. While in most cases it is an incidental finding encountered during routine repair of an inguinal hernia, in other cases, it might be the reason for acute presentation. We are presenting a case of a six-week-old male who was found intraoperatively to have not only the appendix but also the caecum in the hernia sac. We believe that a preoperative groin ultrasound scan would aid clinical diagnosis and facilitate surgical planning in these cases.

## Introduction

An inguinal hernia is one of the common conditions encountered in infants, which require early diagnosis and prompt management due to the risk of incarceration and strangulation that can lead to significant morbidities [1]. Claudius Amyand was a French surgeon who first described the presence of the appendix in the hernia sac in a patient who had appendicitis in 1735 [2]. While the classic definition of Amyand's hernia is the presence of the appendix in the hernia sac [3], other variants include the presence of the appendix as well as the caecum or even the appendix the caecum and the terminal ileum; also, the appendix might be normal, inflamed, incarcerated, strangulated or even perforated. Clinical diagnosis of Amyand's hernia without the aid of imaging modalities can be difficult due to its rarity, as well as the presence of different variants.

## Case presentation

**Figure 1:**
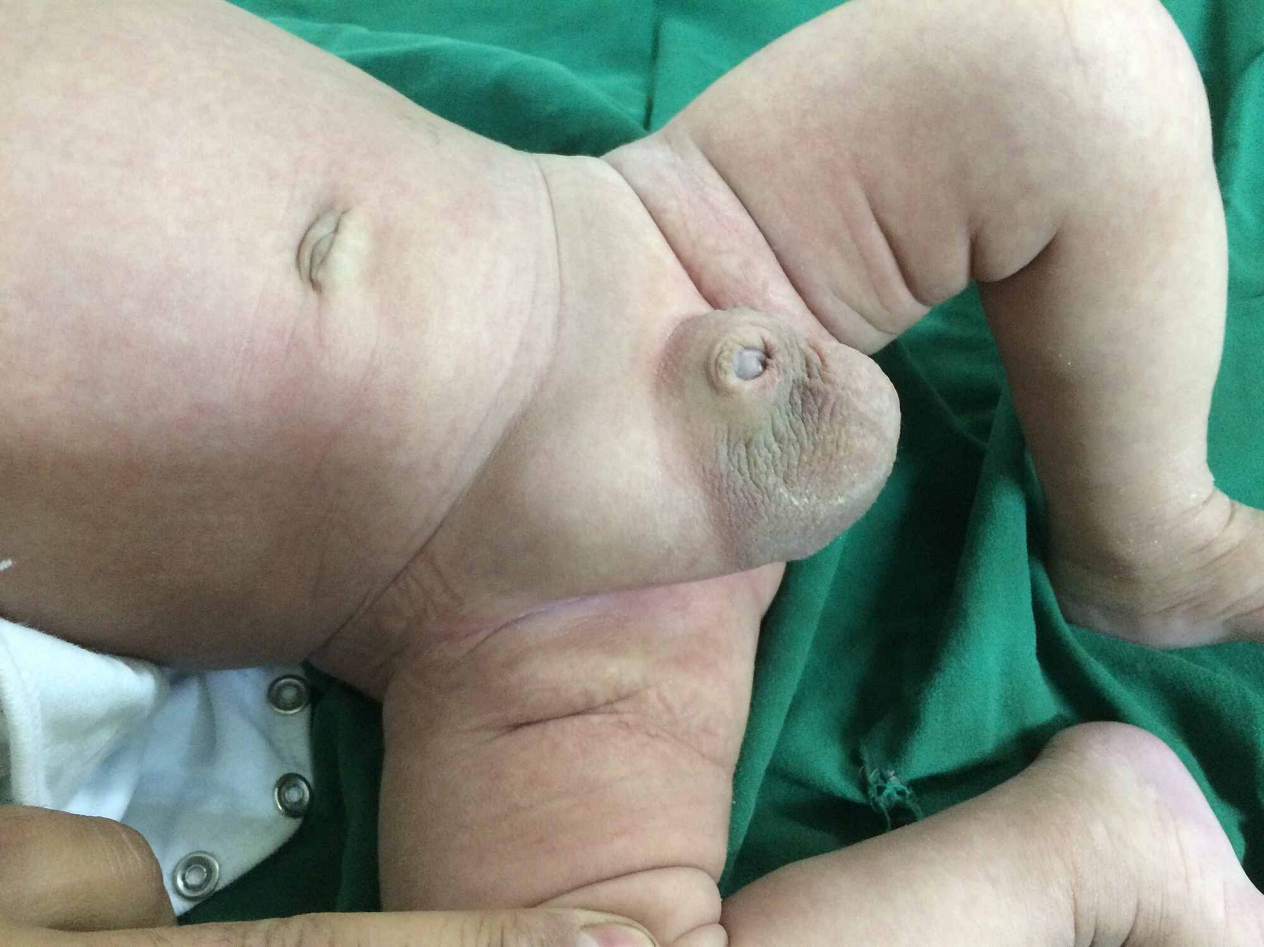
Preoperative picture showing right groin inguinal hernia seen by inspection

We present a case of a six-week-old male infant, born at 39-week gestation, who was brought by his mother to the emergency department with right groin tender swelling, with no history of fever, vomiting, abdominal distension, or diarrhea. His vital signs were normal, and general examination was unremarkable. Abdominal examination revealed no clinical signs of bowel obstruction; groin examination revealed a 40 mm irreducible right-sided inguinal hernia with no signs of strangulation; the scrotal examination was normal (Figure 1).

**Figure 2:**
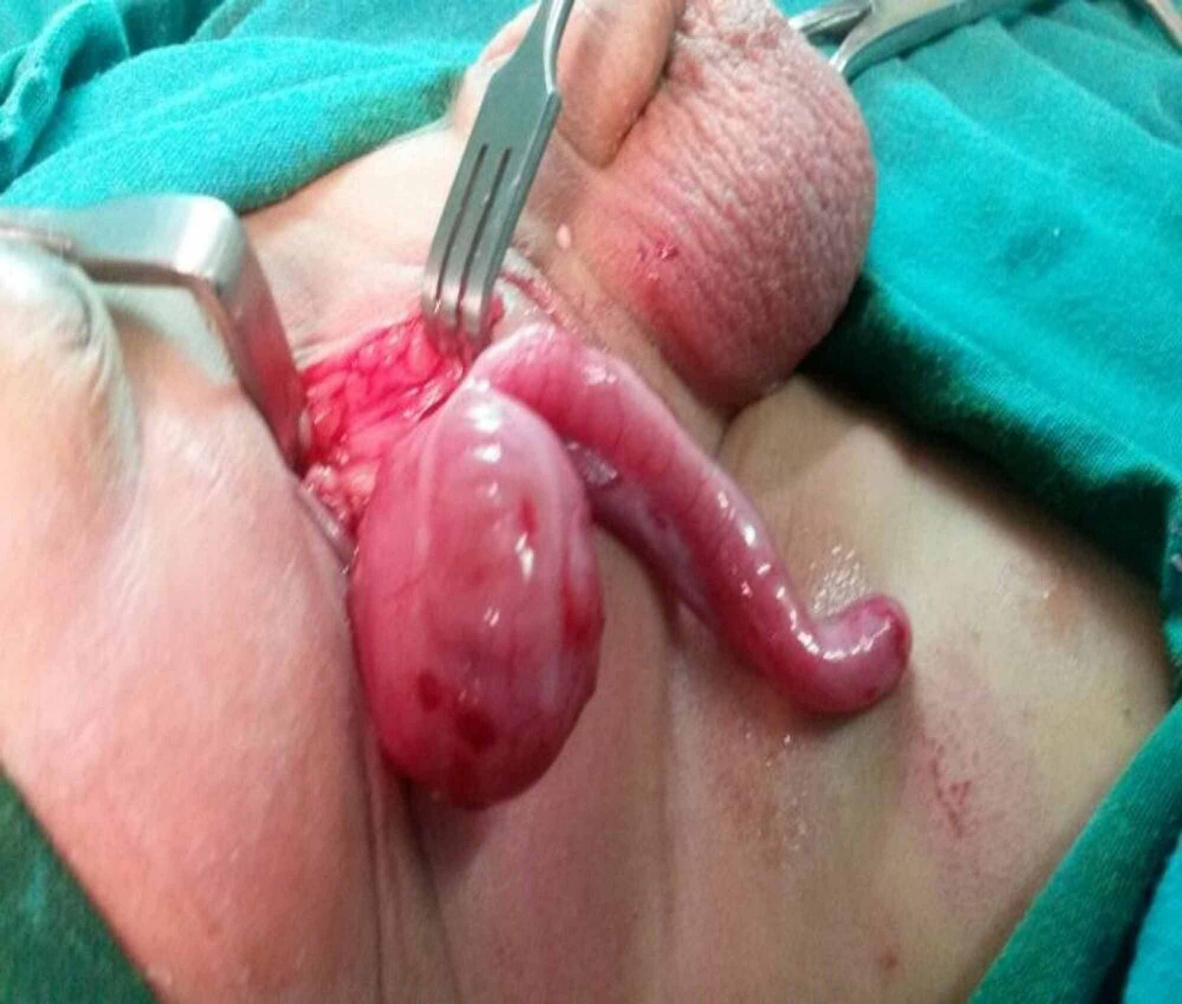
Intraoperative picture showing the caecum and the appendix as part of hernia sac contents

Routine blood tests including full blood count, C-reactive protein (CRP), liver and kidney functions were all within a normal range, and there was no clinical indication for any imaging modality. Our provisional diagnosis was an incarcerated inguinal hernia. Since these types of hernias are better repaired as soon as diagnosed to avoid strangulation, an attempt to repair the hernia through a standard groin incision revealed a viable caecum and long non-inflamed appendix in the hernia sac (Figure 2).

Herniotomy was carried out; the patient had an uneventful recovery, was discharged home the following day, and was seen in the clinic six weeks after, and no signs of postoperative complications were noted.

## Discussion

A congenital inguinal hernia is considered one of the most common surgical diagnoses, with 4% of the pediatric population diagnosed with an inguinal hernia [4], and a cumulative incidence from birth to 15 years of age of 6.62% in males and 0.74 % in females [5]. 

Amyand's hernia is only seen in 1% of all inguinal hernias [3], in most cases reported in males, exclusively on the right side, with exceptions of intestinal malrotation and situs inversus totalis - the conditions reported on the left side [6]. In some rare cases, the appendix can be accompanied by the cecum, omentum, terminal ileum, Meckel diverticulum, urinary bladder, or ovarian and fallopian tube in females [7, 8].

Due to the rarity of the condition and unclear management guidelines, Losanoff and Basson created a classification scale to identify and treat Amyand's hernias based on intraoperative findings [9, 10] (Table 1).

**Table 1:** Losanoff and Basson Amyand's hernia management classification

Classification	Description	Surgical management
Type 1	Normal appendix in an inguinal hernia	Hernia reduction, mesh repair
Type 2	Acute appendicitis in an inguinal hernia, without abdominal sepsis	Appendectomy, primary repair of hernia without mesh
Type 3	Acute appendicitis in an inguinal hernia, with abdominal wall or peritoneal sepsis	Laparotomy, appendectomy, primary repair without mesh
Type 4	Acute appendicitis in an inguinal hernia, with abdominal pathology	Manage as Type 1–3, investigate pathology as needed

Although there has always been an association between prematurity and incidence of strangulation [11], Chang et al., in their large cohort, which comprised 1,000,000 patients found that incarceration was related neither to prematurity nor the waiting time for surgery [5].

CT scan is considered an accurate imaging modality for preoperative diagnosis of Amyand's hernia in adults. Also, an ultrasound scan can show specific features of Amyand's hernia as the presence of a non-compressible, dilated, blind-ending bowel loop with a luminal diameter of more than 7.2 mm, within the inguinal canal with or without surrounding inflammation, which can be used for preoperative diagnosis in pediatric patients [12].

In our case, a type 1 Amyand's hernia was only diagnosed intraoperatively. Given the normal appearance of the caecum and the long appendix, herniotomy was carried out with a good outcome. In hindsight, we think that a quick inguinal ultrasound scan would have provided more insight about the condition before the operation. 

## Conclusions

Amyand's hernia is a rare entity, with a good range of variant clinical presentations and management. Clinical diagnosis without the aid of imaging modalities is not always accurate; therefore, we recommend performing an ultrasound scan at presentation to give an idea about the hernia sac content as well as possible strangulation, which will aid surgical planning.
